# Peroxisome Proliferator-Activated Receptor Agonists: Do They Increase Cardiovascular Risk?

**DOI:** 10.1155/2009/460764

**Published:** 2009-08-19

**Authors:** Ahmad Aljada, Kshitij Ashwin Shah, Shaker A. Mousa

**Affiliations:** ^1^Department of Biomedical Sciences, Long Island University-C.W.POST, Brookville, NY 11548, USA; ^2^Pharmaceutical Research Institute, Albany College of Pharmacy, Albany, NY 12144, USA

## Abstract

Cardiovascular disease is a major cause of morbidity and mortality among people with type 2 diabetes mellitus. The peroxisome proliferator-activated receptor (PPAR) agonists have a significant role on glucose and fat metabolism. Thiazolidinediones (TZDs) are predominantly PPAR*γ* agonists, and their primary benefit appears to be the prevention of diabetic complications by improving glycemic control and lipid profile. Recently, the cardiovascular safety of rosiglitazone was brought to center stage following meta analyses and the interim analysis of the RECORD trial. Current evidence points to rosiglitazone having a greater risk of myocardial ischemic events than placebo, metformin, or sulfonylureas. This review article discusses the mechanism of action of PPAR agonists and correlates it with clinical and laboratory outcomes in the published literature. In addition, this review article attempts to discuss some of the molecular mechanisms regarding the association between TZDs therapy and the nontraditional cardiovascular risks.

## 1. Introduction

 Diabetes Mellitus has emerged as a global burden, as World Health Organization (WHO) has estimated a prevalence of more than 300 million people by the year 2025. Diabetes is associated with a 2–4 fold increased risk for developing unstable angina and myocardial infarction (MI) with subsequent increased mortality and morbidity [1, 2]. Endothelial inflammation, dyslipidemia, fluid retention, edema are among the important factors that play a role in increasing cardiovascular risk in patients with diabetes mellitus. 

PPAR agonists have several metabolic activities which can significantly affect cardiovascular risk. Rosiglitazone and pioglitazone are PPAR*γ* agonists that improve glycemic control and have been shown to exert possible cardiovascular benefits. However, recent studies have shown that rosiglitazone is associated with an increased risk of heart failure, acute MI (AMI) and death as a result of cardiovascular complications [3, 4]. PPARs belong to the nuclear receptor superfamily. They are ligand-activated transcription factors and regulate transcription of target genes by forming heterodimers with the retinoid X receptor (RXR) and binding to specific PPAR response elements (PPREs) in the promoter region of target genes [5]. Three receptor isoforms have been identified, PPAR*α*, PPAR*γ*, and PPAR*δ*. They mediate distinct effects on blood vessel wall, liver, adipose tissue and skeletal muscle (Table 1) [6–8].

TZDs decrease insulin resistance, increase peripheral glucose use, reduce hepatic glucose output and as a result improve overall blood glucose control. In addition, PPAR*γ* ligands have beneficial effects on plasma lipids. Both pioglitazone and rosiglitazone increase serum levels of high-density lipoprotein (HDL) and reverse cholesterol transport. Pioglitazone also reduces plasma triglyceride levels markedly [6–9]. Pioglitazone has more favorable effects on triglycerides than rosiglitazone, although the clinical impact of this finding remains unclear and seems insignificant in clinical practice [10, 11]. The mechanisms underlying differential effects of pioglitazone and rosiglitazone on serum lipids may derive from different degrees of selectivity for PPAR*γ*. Rosiglitazone acts more selectively as a PPAR*γ* agonist while pioglitazone has some additional PPAR*α* agonist activity [12–14]. The differences between pioglitazone and rosiglitazone remain unknown, probably due to mechanisms related both to kinetic properties and pleiotropism of these molecules. Further studies are needed to better understand the mechanisms underlying differential effects of these drugs on lipid metabolism and the meaning of these effects in terms of cardiovascular prevention. PPAR “off-target” signalling remains a possibility and requires further elucidation.

## 2. Effects of PPAR Agonists on Cardiovascular Events

Pioglitazone Effect on Regression of Intravascular Sonographic Coronary Obstruction Prospective Evaluation (PERISCOPE) was designed to compare the effects of Glimepride with pioglitazone on atherosclerosis in patients with type 2 diabetes. It showed that pioglitazone significantly lowered the rate of progression of atherosclerosis in these patients [15]. Similarly, the PROactive study (PROspective pioglitAzone Clinical Trial In macroVascular Events) is a large-scale prospective clinical trial examining the effects of TZDs on cardiovascular outcomes. In this placebo-controlled study of 5238 patients with type 2 diabetes and significant macrovascular disease at baseline, patients were randomized to pioglitazone-treated or placebo groups. In this study, pioglitazone significantly reduced a secondary end point (all-cause mortality, nonfatal myocardial infarction (MI), and stroke; hazard ratio, 0.84; *P* = .03) [16]. The pioglitazone-treated group also showed significant changes in lipid profile (increased HDL cholesterol and LDL cholesterol and decreased triglycerides). However, in PROactive study, pioglitazone increased the incidence of heart failure with a relative risk of 1.5% (*P* = .007). Hence, potential unwanted cardiac effects require caution and further rigorous clinical evaluation, because they may have masked any benefit from actions on lipid profile and inflammation. Similarly, the results of an interim analysis of the Rosiglitazone Evaluated for Cardiac Outcomes and Regulation of Glycemia in Diabetes (RECORD) study which was designed to evaluate the effect of rosiglitazone on cardiovascular morbidity and death in approximately 4500 patients showed a significant risk of heart failure (hazard ratio 2.15; with a 95% confidence interval from 1.30 to 3.57, *P* = .003) in those assigned to rosiglitazone group compared with metformin and sulfonylurea. It did not show any increase in AMI [17, 18]. 

**Figure 1:**
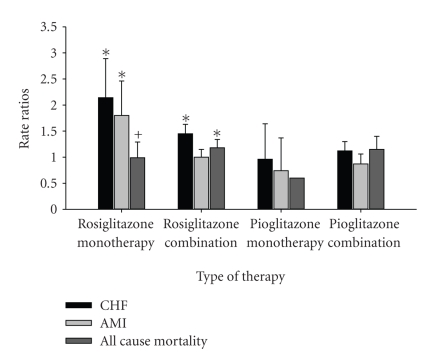
Hazard ratio of chronic heart failure (CHF), acute myocardial infarction (AMI) and all cause mortality for use of TZDs versus other oral hypoglycemic drugs (95% CI). This figure shows the association of TZDs with CHF, AMI and mortality, compared with other oral hypoglycemic agents; *P < .001; ^+^ *P* < .01 [19].

**Figure 2:**
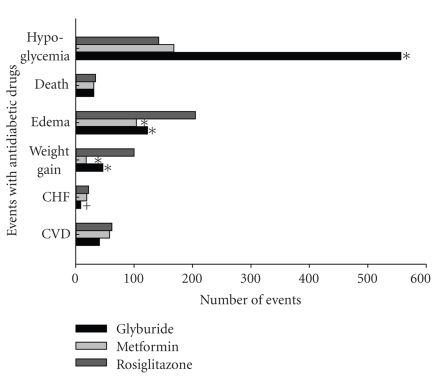
Number of events with antidiabetic drugs. The figure shows the effect of antidiabetic drugs (rosiglitazone, metformin, and glyburide) on various cardiovascular adverse events; *P < .01; ^+^ *P* < .05 for the comparison between metformin and glyburide treatment groups and the rosiglitazone group. The figure is developed from the data of adverse events shown in the Diabetes Outcome Progression Trial (ADOPT) trial [7].

Patients on TZD monotherapy as well as or in combination with other agents were at increased risk of chronic heart failure (CHF). This increased risk was identified only with rosiglitazone. A significant association with CHF risk remained for patients treated with rosiglitazone even among patients with no history of CHF. In addition, TZDs treatment, appeared to be limited to rosiglitazone, was associated with an increased risk of AMI versus users of other oral hypoglycemic agent combinations. Both rosiglitazone monotherapy and combination therapy were associated with an increased risk of death compared with other oral hypoglycemic agent combination therapies (Figure 1) [19]. Similarly, results from the Diabetes Outcome Progression Trial (ADOPT) showed differences in the adverse events among patients receiving monotherapy with rosiglitazone; metformin; glyburide although there was no significant difference in the overall mortality between the three groups. Rosiglitazone group was also significantly associated with edema and the use of loop diuretics than was either metformin or glyburide and higher levels of LDL cholesterol although the death rates were similar, there were other statistically significant differences in adverse outcomes within the three drugs. Rosiglitazone was significantly associated with CHF, edema and raised LDL compared to metformin and glyburide (Figure 2). Various studies compared the differences in the treatment outcomes of pioglitazone and rosiglitazone (Table 2) [19–23]. Broadly, pioglitazone has a better overall clinical and laboratory outcome as compared to rosiglitazone. However, a common limitation was a high drop-out rate of patients in these studies. 

The adverse effects of full PPAR*γ* agonists have reinforced the need to identify additional therapies that improve insulin sensitivity and treat hyperlipidemia in addition to lowering blood pressure. In this regard, several lines of clinical evidence support the use of two Angiotensin receptor blockers (ARBs), telmisartan and irbesartan, in treating hyperlipidemia and insulin resistance [24]. In the Ongoing Telmisartan Alone and in Combination with Ramipril Global Endpoint Trial (ONTARGET), subjects with increased risk for cardiovascular events were randomized to receive telmisartan, ramipril, or a combination of telmisartan and ramipril, while in the companion Telmisartan Randomized Assessment Study in ACE-Intolerant Subjects with Cardiovascular Disease (TRANSCEND) trial, subjects intolerant to ACE inhibitors were randomized to telmisartan or placebo [25]. This study included 5926 participants who were randomly assigned to telmisartan 80 mg/day (*n* = 2954) or placebo (*n* = 2972). The primary endpoint was the composite of cardiovascular death, MI, stroke or hospitalization due to heart failure. The secondary outcome excluded heart failure. Median follow-up was 56 months [26]. There was no difference in the primary composite endpoint of cardiovascular death, myocardial infarction, stroke, or admission to hospital for heart failure. These studies indicate that Angiotensin converting enzyme inhibitors (ACEi) will probably remain the first choice due to the greater body of supportive evidence.

## 3. PPAR*α* and Cardiovascular Events

PPAR*α* by regulating the expression of proteins involved in the transport and *β*-oxidation of free fatty acids (FFAs) plays a pivotal role in the regulation of lipid and glucose metabolism [27]. Fibrates, widely used to treat hypertriglyceridemia, are weak activators of PPAR*α*. They lower circulating triglyceride levels by increasing the activity of lipoprotein lipase (LPL) which hydrolyzes triglycerides [28]. PPAR*α* agonists increase the gene expresson of LPL and up regulate Apo A-I and A-II synthesis which are major apoproteins of the HDL fraction in the liver and resulting in increased serum HDL levels [29, 30]. Data from large clinical trials suggested that fibrates reduce cardiovascular risk, particularly in high-risk populations. In the Veterans Affairs High-Density Lipoprotein InterventionTrial (VA-HIT), gemfibrozil significantly decreased coronary heart disease (CHD) mortality by 41% as compared to those patients with diabetes mellitus receiving the standard treatment [31]. In the Helsinki Heart Study (HHS), gemfibrozil reduced coronary risk by 34% in the overall study population. Coronary artery disease (CAD) events occurred in 3.4% and 10.5% of gemfibrozil and placebo treated patients with diabetes, respectively, although this difference did not achieve statistical significance [32, 33]. Similarly, Fenofibrate Intervention and Event Lowering in Diabetes (FIELD) study investigated the effects of fenofibrate on cardiovascular risk in 9795 patients with type 2 diabetes. Fenofibrate caused an 11% reduction in total cardiovascular events. These studies suggest that PPAR*α* agonists are possibly beneficial in the clinical scenario [34]. 

Several studies have shown that pioglitazone increased serum HDL-cholesterol and decreased triglycerides, and pioglitazone produced more favorable lipid profiles than rosiglitazone in patients with type 2 diabetes mellitus [13, 35]. Notably, Szapary et al. have shown that pioglitazone treatment for 12 weeks significantly increased Apo-AII by 7.7% [35] while Qin et al. demonstrated that pioglitazone stimulates Apo-AI production in HepG2 cells by through PPAR*α* activation [12]. They have also shown that pioglitazone increases apoA-II synthesis and mRNA expression in HepG2 cells. These findings support the notion that pioglitazone increases apoA-I and apoA-II through its PPAR*α* binding.

## 4. TZDs and Nonconventional Cardiovascular Risk Factors

To establish a possible role of pioglitazone and rosiglitazone in prevention of cardiovascular disease appears the primary issue in clinical practice. A novel interest is developing in the so-called nonconventional cardiovascular risk factors including inflammation, homocysteine (HCT) and lipoprotein (Lp(a)). In recent years it has been established that inflammation has a pathogenic role in atherosclerosis. Several studies described the antiinflammatory properties of PPAR*γ* and PPAR*α* agonists which ultimately inhibit atherosclerosis by decreasing the expression of several inflammatory mediators involved in macrophage activation and vascular smooth muscle cells (VSMCs) proliferation (Table 3). Again, the possibility of PPAR “off-target” signaling exists in several of these studies describing the antiinflammatory effects of PPAR. A clear distinction whether these properties are mediated through PPAR*γ* or PPAR*α* was not established as well in many of these studies described in Table 3. Several studies have suggested a possible predictive association between Lp(a), thoracic aortic atherosclerosis and stroke. Hyperhomocysteinemia seems to be an independent factor for atherothrombotic events both in diabetic and nondiabetic patients. Treatment with pioglitazone in subjects with type 2 diabetes and metabolic syndrome for 12 months provided a significant decrease in Lp(a) concentration despite a substantial neutrality of rosiglitazone plus metformin combination; HCT significantly decreased in the rosiglitazone plus metformin group after 12 months [36]. Similarly, rosiglitazone reduced Lp(a) despite a significant increase in fibrinogen [37].

## 5. Future Studies

The cardiovascular benefit-risk ratio of individual PPAR agonists is not completely clear. The results of data analysis of the RECORD study showed an increased risk of heart failure in the rosiglitazone group compared with metformin and sulfonylurea. It did not show any increase in AMI [17, 18]. These results suggest increased cardio toxic effects with the use of rosiglitazone. Furthermore, any potential antiatherosclerotic benefits must be weighed against the increased risk of CHF. Several ongoing studies may provide more information including the Bypass Angioplasty Revascularization Investigation 2 Diabetes (BARI-2D) which is a randomized trial to study comparing insulin-stimulating medication versus medication that sensitizes the body to available insulin in patients with type 2 diabetes and coronary artery disease [59]. Another ongoing study is the Action to Control Cardiovascular Risk in Diabetes (ACCORD). This is a clinical trial of patients with type 2 diabetes whose baseline HbA1c levels are less than 7.5% and at high risk for CVD events due to CVD risk factors or previous CVD events. The ACCORD study is examining whether aggressive glucose lowering using a variety of strategies prevents CVD events in patients with type 2 diabetes in order to support future clinical guidelines for diabetes management in older adults. Currently, no PPAR*δ* agonists are clinically approved at the present moment, but they may be beneficial for the treatment of cardiovascular disorders and improve overall cardiovascular risk assessment. 

Ongoing Phase II clinical trials of GW-501516, a PPAR*δ* agonist for the potential treatment of dyslipidemia by GlaxoSmithKline and Ligand are soon to be released. GW-501516 may prove to be a suitable alternative for the treatment of the cardiovascular disease and improve overall cardiovascular risk assessment. Interestingly, GW501516 suppresses IL-6-mediated hepatocyte acute phase reaction via STAT3 inhibition [60].

## 6. Conclusions

The findings reviewed in this article suggest that the mechanisms of PPAR agonists have an excellent glucose and lipid control by their effects on vasculature, muscle and adipose tissue. The in vitro studies also show their effects by improving lipid profile, maintaining euglycemia, suppressing various inflammatory mediators, HCT, Lp(a), and preventing the progression of atherosclerosis. However, when clinical trials are reviewed, they do not reflect very well with the beneficial actions in cell and animal models. Rosiglitazone has shown adverse effects on heart failure, mortality, and abnormal lipid profile. Pioglitazone is associated with increased risk of heart failure due to edema. Furthermore, pioglitazone has been shown to reduce the cardiovascular risk in patients with established atherosclerotic vascular disease. Pioglitazone has also a favorable effect on lipid profile by stimulating apoA1 production because it has some additional PPAR*α* activity. A larger population study is needed to understand PPAR agonists antiatherogenic properties. However, their adverse effects should be taken into considerations.

## Figures and Tables

**Table 1:** Effects of PPAR*α* and PPAR*γ* on vessel wall, liver, adipose and muscle tissues [6–8].

	Vessel wall	Liver	Adipose	Muscle
PPAR*α*	↓ Inflammation Reverses cholesterol transport	↓ Triglyceride ↑ HDL; ↓ Small dense LDL	↑ Adipogenesis	↑ FFA oxidation

PPAR*γ*	↓ Inflammation Reverses cholesterol transport	↑ fatty acid storage	↑ Adiponectin ↑ Fatty acid storage	↑ Glucose uptake

**Table 2:** The effect of TZDs on CV risk. The table shows a comparison between rosiglitazone and pioglitazone on lipid profile and HbA1c levels in various studies [19–23].

TZDs versus placebo	Pioglitazone	Rosiglitazone
Total cholesterol	Neutral	Raised
LDL	Neutral	Raised
HDL	Raised	Raised
Triglycerides	Reduced	Neutral
HbA1c	↓ 1–1.5%	↓ 1–1.5%

**Table 3:** Effects of PPAR*γ* and PPAR*α* agonists on inflammatory mediators. Table shows the effect of PPAR*γ* and PPAR*α* receptors modulation on the expression of various inflammatory mediators. ICAM: intercellular adhesion molecule; VCAM: vascular cell adhesion molecule; IL: Interleukin; TNF-*α*: Tumor necrosis factor-*α*; MMP-9: Matrix metalloproteinase-9; IFN: interferon; NF: Nuclear factor; iNOS: Inducible nitric oxide synthase; CRP: C-Reactive protein; CD40L: CD40 Ligand; LOX-1: low-density lipoprotein receptor-1; AP-1: Activator protein-1 [29, 30, 38–58].

	PPAR*γ* agonists reduce/decrease	PPAR*α* agonists reduce/decrease
Endothelium	ICAM, VCAM, superoxide generation	ICAM, VCAM, E-selectin
Macrophage	IL-1, IL-2, TNF*α*, MMP-9, IFN-*γ*, NF*κ*B, iNOS	IL-1, IL-2, TNF*α*, MMP-9, IFN-*γ*
Serum/Plasma	CRP, MMP-9, IL-6, soluble CD40L	IL-6, Fibrinogen, CRP, MIF
Vascular smooth muscle cells	VEGF, MMP-9, IL-1*β*, IL-6, TGF*β*, LOX-1, TNF*α*	IL-1*β*, IL-6, fibrinogen-*β*, prostaglandin, NF*κ*B, AP-1
